# Leptin and leptin receptor gene polymorphisms and depression treatment response

**DOI:** 10.1017/neu.2024.43

**Published:** 2024-11-12

**Authors:** Ida-Maria Tavast, Anssi Solismaa, Leo-Pekka Lyytikäinen, Nina Mononen, Eeva Moilanen, Mari Hämäläinen, Terho Lehtimäki, Olli Kampman

**Affiliations:** 1 Department of Psychiatry, Faculty of Medicine and Health Technology, Tampere University, Tampere, Finland; 2 Department of Psychiatry, The Pirkanmaa Wellbeing Services County, Tampere, Finland; 3 Department of Clinical Chemistry, Tampere University Hospital and Fimlab Laboratories and Finnish Cardiovascular Research Center-Tampere, Faculty of Medicine and Health Technology, Tampere University, Tampere, Finland; 4 The Immunopharmacology Research Group, Faculty of Medicine and Health Technology, Tampere University and Tampere University Hospital, Tampere, Finland; 5 Department of Psychiatry, Department of Clinical Sciences (Psychiatry), Faculty of Medicine, University Hospital of Umeå, Umeå University, Umeå, Sweden; 6 Department of Clinical Medicine (Psychiatry), Faculty of Medicine, University of Turku, Turku, Finland; 7 Department of Psychiatry, The Wellbeing Services County of Ostrobothnia, Vaasa, Finland

**Keywords:** Depression, genetics, leptin, leptin receptor, treatment outcome

## Abstract

**Objective:**

Associations between leptin (*LEP*) and leptin receptor (*LEPR*) gene polymorphisms and mood disorders have been found but not yet confirmed in multiple studies. The aim of our study was to study the associations between *LEP* and *LEPR* single nucleotide polymorphisms (SNPs) and treatment response of depression. Associations between leptin levels and depression severity were also investigated.

**Methods:**

The data included 242 depressed patients in secondary psychiatric care. Symptoms of depression were assessed with the Montgomery–Åsberg Depression Rating Scale (MADRS). Previously found *LEP* and *LEPR* SNPs associated with depression and other mood disorders were studied. Furthermore, all available *LEP* and *LEPR* SNPs were clumped using proxy SNPs to represent gene areas in *r*^2^ > 0.2 linkage disequilibrium and their association with treatment response was analysed with logistic regression.

**Results:**

Two proxy SNPs of *LEPR* gene, rs12564738 and rs12029311, were associated with MADRS response at 6 weeks (*p* adjusted = 0.024, *p* adjusted = 0.024). SNPs from previous studies were not associated with MADRS response, but *LEPR* rs12145690 from a previous study was strongly associated with rs12564738 (*r*^2^ = 0.94). The positive association between leptin levels and MADRS score at baseline after adjusting with age, sex, body mass index (BMI), Alcohol Use Disorders Identification Test score, and smoking was found (*p* = 0.011).

**Conclusion:**

Our findings suggest that *LEPR* polymorphisms are associated with depression treatment response. We also found associations between leptin levels and depression independently of BMI. Further studies and meta-analyses are needed to confirm the significance of found SNPs and the role of leptin in depression.

## Graphical abstract

**Figure:**
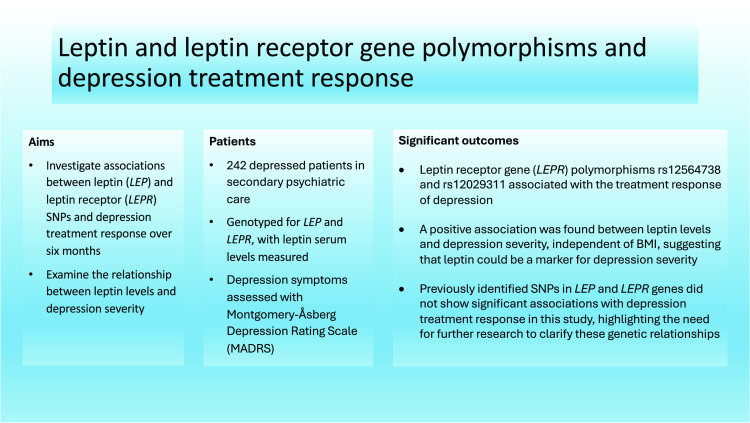

Highlights

- The study identified significant associations between leptin receptor gene (*LEPR*) polymorphisms rs12564738 and rs12029311 and the treatment response of depression, indicating their potential role in predicting treatment outcomes.
- A positive association was found between leptin levels and depression severity, independent of body mass index (BMI), suggesting that leptin could be a marker for depression severity.
- Previously identified single nucleotide polymorphisms in *LEP* and *LEPR* genes did not show significant associations with depression treatment response in this study, highlighting the need for further research to clarify these genetic relationships.

Significant outcomes

- *LEPR* polymorphisms rs12564738 and rs12029311 are associated with depression treatment response.
- An association between leptin levels and depression severity was found independently of BMI.

Limitations

- Sample size was relatively limited.
- The sample included patients with harmful alcohol use.
- There was variation in the onset of depression and previous treatments between patients.

## Introduction

Major depressive disorder (MDD) is a common psychiatric illness with a global prevalence of around 5 % (Ferrari *et al*., 2013). It is one of the most significant contributors to disability globally (Atun, 2015). Depression is a multifactorial disorder, and its ethiology and pathophysiology are not fully understood. Depression is associated with obesity, and they may have shared biological mechanisms, such as the dysregulation of leptin (Milaneschi *et al*., 2019).

Leptin is a 167 amino acid protein (16 kDa) encoded by the leptin gene (*LEP)* on chromosome 7 and primarily secreted from white adipose tissue in proportion to the percentage of body fat (Münzberg & Morrison, 2015). Leptin is best known for its role in the regulation of energy homeostasis by reducing energy intake and increasing energy consumption (Farr *et al*., 2015). Leptin binds to leptin receptors (*LEPR*) encoded by the *LEPR* gene on chromosome 1 (Endomba *et al*., 2020). Leptin receptors are located in different parts of the body, including the central nervous system, where leptin receptors have been found in hypothalamic and thalamic regions, hippocampus, amygdala, substantia nigra, cortex, brainstem, cerebellum, and some other brain regions (Erichsen *et al*., 2022).

In the central nervous system, leptin modulates stress adaptation and the control of energy homeostasis via the hypothalamic-pituitary-adrenal (HPA) axis (Roubos *et al*., 2012). Hyperactivation of the HPA axis is associated with MDD (Pariante & Lightman, 2008). Leptin also regulates neural plasticity, especially in brain regions related to depression, by affecting neuronal morphology and hippocampus synaptic transmission and by working as a neurotrophic factor via brain-derived neurotrophic factor (Ge *et al*., 2018), which has been linked to depression and antidepressant effects (Castrén & Monteggia, 2021). In animal studies, leptin has reduced depressive behaviour caused by chronic unpredictable stress and had antidepressant effects (Lu *et al*., 2006; Garza *et al*., 2012). However, studies with humans have thus far been controversial reporting both increased and decreased leptin levels in depressed patients (Zou *et al*., 2019); thus, leptin has not been identified as a biomarker for depression. Furthermore, acute alcohol use reduces plasma leptin levels, whereas chronic alcohol use may increase leptin levels (Bach *et al*., 2021). Smoking is associated with lower leptin levels (Shaheen *et al*., 2023).

Leptin resistance has also been suggested to be associated with depression and explain the relationship between depression and obesity (Lu, 2007; Yamada *et al*., 2011). *LEP* and *LEPR* polymorphisms are one factor that can cause leptin resistance (Liu *et al*., 2018). Mutations in the *LEP* gene may also lead to a reduced leptin levels, as well as alterations in its production (Socol *et al*., 2022). Some genetic studies have investigated the association between leptin polymorphism and addictions, depression, and other mental disorders, but the impact of these polymorphisms has not yet been verified. Short variants (<208bp) of the *LEP* gene D7S1875 short tandem repeat marker are potential risk factors for depression (Kapoor *et al*., 2009). *LEPR* gene variant rs1137101 is linked to high leptin concentrations but no association has been found between the *LEPR* gene variant rs1137101 and depressive symptoms (Reis *et al*., 2015). Certain genetic polymorphisms of the leptin gene (rs10487506, rs4731423, rs2278815, rs4731426, rs12706832, rs11763517, and rs3828942) are risk factors for antidepressant treatment resistance in depressed patients (Kloiber *et al*., 2013). In addition, an association has been found between the *LEPR* gene rs1171276 variant and a higher suicide risk (Acikel *et al*., 2020), as well as between *LEPR* gene variants (rs1137100, rs12145690, rs8179183) and better treatment response in bipolar disorder (Chang *et al*., 2022).

The aim of this study is to study associations between depression treatment response and *LEP* and *LEPR* variants by analysing all available single nucleotide polymorphism (SNP) variations in these genes and examining the polymorphisms found in previous studies separately. The secondary aim was to investigate the association between leptin levels and depression severity. These findings could help in developing tools to predict treatment response in depression and provide new information about the genetic mechanisms between leptin and depression.

## Patients and methods

### Study design and participants

The study protocol is registered in ClinicalTrials.gov with an identifier NCT02520271 (Ostrobothnia Depression Study [ODS], 2016). From 2009 to 2013, 242 patients were included in the study from one psychiatric hospital ward and from five psychiatric outpatient clinics located in the South Ostrobothnia Hospital District of Finland with a population of 200,000. Patients had depressive symptoms and comorbid problems such as anxiety, self-destructiveness, insomnia, or alcohol or other substance use. Beck Depression Inventory (BDI, version 1A) (Beck *et al*., 1996) was used to screen patients and at least moderately depressed patients were recruited (BDI score ≥17). Two-thirds of patients had a recurrent depression. Patients who had primary psychotic disorders (ICD-10 codes F20-29) or organic brain disease or brain damage were excluded from the study. The local ethics committee approved the study, and all participants gave a written informed consent.

Diagnostic and symptom assessments were performed at baseline and included Mini International Neuropsychiatric Interview 5.0 (MINI) (Sheehan *et al*., 1998), BDI-21 and Montgomery–Åsberg Depression Rating Scale (MADRS) (Montgomery & Asberg, 1979). Alcohol use was evaluated using the Alcohol Use Disorders Identification Test (AUDIT) (Babor *et al*., 2001) and timeline follow-back (Sobell *et al*., 1986) about alcohol amounts per week and duration of harmful drinking. Other substance use during the past 12 months and self-reported smoking status were also assessed. Blood samples were collected for laboratory tests. The patients were weighted, and their length was measured by a study nurse.

All patients received Behavioural Activation Therapy (Cuijpers *et al*., 2023) for up to 6 months, and patients with substance abuse received additional motivational interview. The study protocol did not provide specific instructions for medication except medication dosing regimen if MADRS score was over 19 points at baseline. In the follow-up visits at 6 weeks and 6 months, the severity of depression was assessed with MADRS, and alcohol use was assessed with AUDIT. Laboratory tests were measured at baseline and at 6 months. Treatment response was determined as at least a 50% reduction with the MADRS scale (Nierenberg & DeCecco, 2001).

### Laboratory analyses

Venous blood samples were collected from each patient at baseline and at 6 months. All samples were collected in the morning to control for daily changes in circulating leptin. The serum was centrifugated and stored at the temperature of –80°C before the analysis. Leptin levels were measured by enzyme-linked immunosorbent assay with commercial reagents (R&D Systems, Abingdon Science Park, UK). The lowest standard and inter-assay coefficient of variation was 15.6 pg/ml and 3.6% for leptin.

### Genotyping and imputation

QIAamp DNA Blood Midikit and an automated biorobot M48 extraction (Qiagen, Hilden, Germany) were used to extract genomic DNA from peripheral blood leukocytes. The genotyping of samples was performed by using an Illumina Infinium HumanCoreExome-12 DNA Analysis Beadchip version 1, following the manufacturer’s instructions at Helmholtz Zentrum, München, Germany. The quality was controlled by the following filters: GenTrain score < 0.20, GenCall score < 0.15, sample and an SNP call rate <0.95, excess heterozygosity, Hardy–Weinberg equation *P* value < 10^−6^, cryptic relatedness (pi-hat > 0.2), multidimensional scaling, and gender check. Imputation was done by using SHAPEIT v2 in haplotype phasing and IMPUTE2 v.2.3.2 and 1000 Genomes Phase I integrated variant set haplotypes as a reference in genotype imputation. Well-imputed SNPs had info ≥ 0.3.

### Statistical methods

The distribution of the variables was explored using the normal Q–Q plot and Kolmogorov–Smirnov test. Two-variable associations between age, baseline MADRS, sex, smoking, baseline AUDIT score, and body mass index (BMI) were explored with correlation tests, *t*-tests, and Mann–Whitney *U*-tests. Associations between baseline MADRS and baseline leptin serum levels were analysed with Pearson correlation tests and linear regression models. Leptin serum level distribution was skewed; therefore, a logarithmic transformation was also used. We conducted chi-squared tests to examine differences in treatment response rates by gender at 6 weeks and 6 months. For changes in MADRS scores, we used the Mann–Whitney *U*-test at 6 weeks due to non-normal distribution and a *t*-test at 6 months for normally distributed data. The correlations between independent variables used in the regression models were tested with Spearman correlation coefficients. The variation in serum leptin level was analysed with a linear regression model with age, baseline MADRS, sex, smoking, baseline AUDIT score and BMI as explanatory variables.

Based on a literature search on SNPs in *LEP* and *LEPR* related to depression with the following terms: “leptin” and “depression” and “genetics”, statistically significant SNPs found in the earlier studies were selected. The following SNPs from the *LEP* gene were included in this study: rs10487506, rs4731423, rs2278815, rs4731426, rs12706832, rs11763517, and rs3828942 and SNPs from the *LEPR* gene: rs1171276, rs1137100, rs12145690, and rs8179183 (Kloiber *et al*., 2013; Acikel *et al*., 2020; Chang *et al*., 2022). The association of these SNPs with treatment response was studied using logistic regression and linear regression models, with MADRS response and MADRS score change, respectively, at 6 weeks and 6 months as dependent variables and age, sex, BMI, baseline AUDIT score, smoking, and selected SNPs separately as covariables. As the original studies did not account for alcohol use, the same analyses were also performed excluding patients with AUDIT > 10. To account for multiple testing, *p*-values were adjusted with false discovery rate (FDR).

Lastly, genetic associations between treatment response and all available *LEP* and *LEPR* SNPs were studied. Proxy SNPs were calculated to represent gene areas in r^2^ > 0.2 linkage disequilibrium. Analyses were performed with both logistic regression and linear regression models, with MADRS response and MADRS score change, respectively, at different timepoints as dependent variables and age, sex, BMI, baseline AUDIT score, smoking, and SNPs one at a time as covariables. *P*-values were FDR-adjusted. For assessing collinearity between explanatory variables, a threshold of *r* ≥ 0.5 was used.

All analyses were performed with R version 4.1.1 (R Core Team, 2021).

## Results

Characteristics of the study population are presented in Table 1. Baseline AUDIT score and smoking were correlated significantly (Spearman *r* = 0.39, *p* < 0.0001). Men had markedly higher baseline AUDIT score compared to women (15.1 ± 10.6 vs. 7.93 ± 8.30, Mann–Whitney *U*-test *p* < 0.0001). Men had slightly higher baseline MADRS scores than women (24.5 ± 6.4 vs. 22.3 ± 6.8, *t*-test *p* = 0.012). Patients who smoked had higher MADRS scores than those who did not (24.4 ± 5.8 vs. 21.2 ± 7.0, *t*-test *p* = 0.0012). There was no difference in smoking between men and women (*χ*^2^ = 2.72, *p* = 0.10). BMI had a strong correlation with serum leptin levels (*r* = 0.72, *p* < 0.0001). Mean serum levels of leptin in women and men were 31.0 ± 24.2 and 10.4 ± 10.9, respectively (*t*-test *p* < 0.0001). Age was not correlated with serum leptin levels (*r* = 0.11, *p* = 0.10). A diagnosis of diabetes was present in 5.8% (*n* = 14) of the patients. There was no significant association between diabetes and baseline MADRS scores (*t*-test, *p* = 0.13) or serum leptin levels (*t*-test, *p* = 0.93). There were no differences in treatment response rates by gender at 6 weeks or 6 months (*χ*^2^, *p* = 0.44 and *p* = 0.60, respectively) nor were there differences in MADRS point reduction by gender at 6 weeks (Mann–Whitney *U*-test, *p* = 0.84) or 6 months (*t*-test, *p* = 0.51).

**Table 1.** Characteristics of the study population

	Baseline	6 weeks	6 months	12 months	24 months
N with MADRS available (% of baseline)	226	182 (80.5 %)	148 (65.5 %)	118 (52.2 %)	66 (29.2 %)
Sex	39.8 % male 60.2 % female	40.1 % male 59.9 % female	37.2 % male 62.8 % female	31.4 % male 68.6 % female	33.3 % male 66.7 % female
Age mean ± SD years	38.57 ± 12.10	39.01 ± 12.16	39.82 ± 12.01	40.02 ± 12.13	41.50 ± 12.57
MADRS score mean ± SD	23.16 ± 6.70	16.99 ± 8.06	13.26 ± 8.73	9.86 ± 7.72	8.05 ± 7.66
BMI mean ± SD kg/m^2^	28.22 ± 6.67		28.90 ± 6.74		
Smoking %	53.4 %		46.9 %	44.9 %	41.5 %
AUDIT mean ± SD	10.71 ± 9.88		7.48 ± 7.41	6.19 ± 6.51	6.45 ± 7.24
Serum leptin level mean ± SD µg/l	22.98 ± 22.44		26.09 ± 22.36		

MADRS, Montgomery–Åsberg Depression Rating Scale; BMI, body mass index; AUDIT, Alcohol Use Disorders Identification Test.

No correlation between baseline MADRS and baseline leptin serum levels was found (Pearson *r* = 0.041, *p* = 0.56). Baseline MADRS score was associated with leptin serum level in a linear regression model adjusted with age, sex, smoking, baseline AUDIT score, and BMI (Table 2). None of the explaining factors used in the model had a correlation (Spearman) of 0.5 or higher with each other.

**Table 2.** Linear regression model with leptin serum level as dependent variable and age, baseline MADRS score, sex, smoking, baseline AUDIT score and BMI as covariables

	B	z	p
Age	−0.0041	−1.086	0.28
MADRS (baseline)	0.020*	2.59	**0.011**
Sex (female)	1.13	11.57	**<0.001**
Smoking (yes)	−0.20	−2.010	**0.047**
AUDIT (baseline)	0.0040	0.78	0.44
BMI	0.12	16.40	**<0.001**

MADRS, Montgomery–Åsberg Depression Rating Scale; AUDIT, Alcohol Use Disorders Identification Test; BMI, body mass index.

*Positive estimate (B) means that patients with higher MADRS score had a higher serum leptin level.

In the logistic regression model explaining MADRS response at 6 weeks and at 6 months with age, sex, BMI, AUDIT score, smoking, and selected SNPs from previous studies as covariables, no SNP was statistically significant after adjusting the *p*-value (Tables 3 and 4). Due to the strong correlation between BMI and leptin levels, leptin was not included in the regression analysis to avoid multicollinearity. Excluding patients with AUDIT score > 10 had no substantial effect on the results.

**Table 3.** Logistic regression model explaining MADRS response at 6 weeks and at 6 months with age, sex, BMI, AUDIT score, smoking, and selected SNPs from previous studies as covariables

		MADRS response at 6 weeks				MADRS response at 6 months			
	Previous publication	B	*t*	*p*	p adj.*	B	*t*	*p*	p adj.*
rs10487506	(Kloiber *et al*., 2013)	0.25	0.68	0.49	0.72	−0.073	−0.20	0.84	0.97
rs4731423	(Kloiber *et al*., 2013)	0.37	1.04	0.30	0.69	0.20	0.56	0.57	0.90
rs2278815	(Kloiber *et al*., 2013)	0.36	1.01	0.31	0.69	0.21	0.61	0.54	0.90
rs4731426	(Kloiber *et al*., 2013)	0.36	1.01	0.31	0.69	0.21	0.61	0.54	0.90
rs12706832	(Kloiber *et al*., 2013)	0.19	0.54	0.59	0.72	0.31	0.91	0.36	0.90
rs11763517	(Kloiber *et al*., 2013)	0.23	0.56	0.58	0.72	0.47	1.19	0.23	0.90
rs3828942	(Kloiber *et al*., 2013)	−0.089	−0.23	0.82	0.82	−0.15	−0.41	0.68	0.94
rs1171276	(Acikel *et al*., 2020)	−0.34	−0.43	0.66	0.73	−0.14	−0.15	0.88	0.97
rs1137100	(Chang *et al*., 2022)	0.46	1.25	0.21	0.69	0.0083	0.024	0.98	0.98
rs12145690	(Chang *et al*., 2022)	0.75	1.86	0.063	0.69	0.31	0.81	0.42	0.90
rs8179183	(Chang *et al*., 2022)	−0.51	−0.84	0.40	0.72	−1.015	−1.67	0.095	0.90

MADRS, Montgomery–Åsberg Depression Rating Scale.

*FDR-adjusted *p*.

**Table 4.** Logistic regression model explaining MADRS response at 6 weeks and at 6 months with age, sex, BMI, smoking, and selected SNPs from previous studies as covariables excluding patients with AUDIT > 10

		MADRS response at 6 weeks				MADRS response at 6 months			
	Previous publication	B	*t*	*p*	p adj.*	B	*t*	*p*	p adj.*
rs10487506	(Kloiber *et al*., 2013)	0.19	0.56	0.57	0.81	−0.29	−0.93	0.35	0.65
rs4731423	(Kloiber *et al*., 2013)	0.27	0.84	0.40	0.74	−0.014	−0.048	0.96	0.998
rs2278815	(Kloiber *et al*., 2013)	0.26	0.83	0.41	0.74	−0.00091	−0.0030	0.998	0.998
rs4731426	(Kloiber *et al*., 2013)	0.26	0.83	0.41	0.74	−0.00076	−0.0025	0.998	0.998
rs12706832	(Kloiber *et al*., 2013)	0.14	0.43	0.67	0.81	0.042	0.14	0.89	0.998
rs11763517	(Kloiber *et al*., 2013)	0.17	0.48	0.63	0.81	0.74	2.17	**0.030**	0.16
rs3828942	(Kloiber *et al*., 2013)	−0.11	−0.31	0.76	0.84	−0.36	−1.12	0.26	0.58
rs1171276	(Acikel *et al*., 2020)	−0.097	−0.13	0.90	0.90	−0.996	−1.26	0.21	0.58
rs1137100	(Chang *et al*., 2022)	0.30	0.93	0.35	0.74	−0.016	−0.054	0.96	0.998
rs12145690	(Chang *et al*., 2022)	0.60	1.66	0.098	0.74	0.41	1.19	0.24	0.58
rs8179183	(Chang *et al*., 2022)	−0.60	−1.19	0.23	0.74	−1.13	−2.24	**0.025**	0.16

MADRS, Montgomery–Åsberg Depression Rating Scale.

*FDR-adjusted *p*.

In linear regression models explaining MADRS score change at 6 weeks or at 6 months with age, sex, BMI, AUDIT score, smoking, and selected SNPs from previous studies as covariables, no SNP was statistically significant.

**Figure1.**
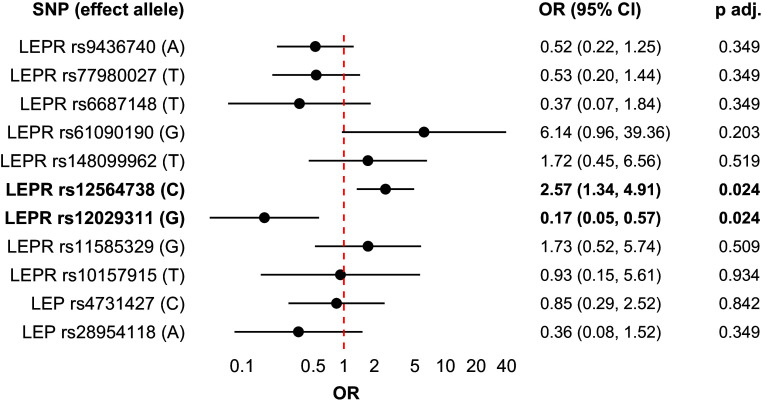
Forest plot of odds ratios (OR) from logistic regression examining treatment response at 6 weeks for clumped LEPR and LEP SNPs, adjusted for age, sex, BMI, AUDIT score, and smoking, including effect alleles and FDR-adjusted *p*-values. OR > 1: for each additional effect allele (going from 0 to 1 or 1 to 2), the odds of treatment response increase by the factor of the OR. OR < 1: for each additional effect allele, the odds of treatment response decrease by the factor of the OR.

When analysing all available LEP and LEPR SNPs, two proxy SNPs of *LEPR* gene, rs12564738 and rs12029311, were associated with MADRS response at 6 weeks in a logistic regression model adjusted with age, sex, BMI, AUDIT score, and smoking (Table 5, Figure 1).

**Table 5.** Proxy SNPs after clumping of all available LEP and LEPR SNPs and their association with MADRS response at 6 weeks in a logistic regression model adjusted with age, sex, BMI, AUDIT score and smoking

SNP	Effect allele	Gene	Location	Chromosome	MAF	B	*z*	*p*	p.adj
rs12564738	C	LEPR	Upstream transcript variant	1	0.44	0.94	2.86	**0.0043**	**0.024**
rs12029311	G	LEPR	Intron variant	1	0.075	−1.80	−2.85	**0.0043**	**0.024**
rs61090190	G	LEPR	Intron variant	1	0.060	1.82	1.92	0.055	0.20
rs9436740	A	LEPR	Intron variant	1	0.23	−0.65	−1.46	0.14	0.35
rs77980027	T	LEPR	Intron variant	1	0.083	−0.63	−1.24	0.22	0.35
rs6687148	T	LEPR	Intron variant	1	0.054	−1.0047	−1.22	0.22	0.35
rs28954118	A	LEP	3’-UTR variant	7	0.051	−1.028	−1.39	0.16	0.35
rs11585329	G	LEPR	Intron variant	1	0.095	0.55	0.90	0.37	0.51
rs148099962	T	LEPR	Intron variant	1	0.11	0.54	0.80	0.42	0.52
rs4731427	C	LEP	Intron variant	7	0.086	−0.17	−0.30	0.77	0.84
rs10157915	T	LEPR	Intron variant	1	0.052	−0.076	−0.083	0.93	0.93

SNP, single nucleotide polymorphism; LEPR, leptin receptor; LEP, leptin.

## Discussion

In this study, two proxy SNPs in *LEPR* gene, rs12564738 and rs12029311, were associated with treatment response at 6 weeks. The significant SNPs from previous studies were not associated with depression treatment response in our study. In addition, we found an association between baseline depression severity and leptin levels after adjusting with age, sex, BMI, AUDIT score, and smoking.

After analysing all available variations in *LEP* and *LEPR* genes, two *LEPR* polymorphisms, rs12564738 and rs12029311, were associated with MADRS response at 6 weeks. There are no previous publications on these polymorphisms. *LEPR* polymorphism rs12564738 is an upstream transcript variant and transforms *C* > G or *C* > T in the upstream of the *LEPR* gene. Rs12029311 is an intron variant of the *LEPR* gene and transforms *G* > A or *G* > T in the *LEPR* gene. These proxy SNPs represent a larger number of polymorphisms, and it is possible that detected effect comes through other polymorphisms connected to these SNPs. We searched previous publications on SNPs that were strongly connected to these proxy SNPs with r^2^ > 0.8. Rs3790435 was associated with metabolically unhealthy obesity in children (Abaturov & Nikulina, 2021) and with obstructive sleep apnoea risk (Li *et al*., 2019). Rs1327118 was associated with a decreased risk of type 2 diabetes in men and increased systolic and diastolic blood pressure and decreased HDL cholesterol levels in women (Zhang *et al*., 2018). Rs12145690 was associated with better treatment response of bipolar disorder (Chang *et al*., 2022) and with plasma leptin levels in women (Ortega-Azorín *et al*., 2019). It was also one of the SNPs we analysed separately in our study. The effect of rs12145690 was the largest compared to other SNPs, but it did not reach statistical significance in our data. However, it was strongly connected (*r*^2^ = 0.94) to proxy SNP rs12564738 which was associated with MADRS response at 6 weeks in our study. Further meta-analyses are needed to confirm the significance of rs12145690.

Based on the literature search, we chose several *LEP* and *LEPR* gene SNPs that have been associated with depression and other psychiatric disorders in previous studies and analysed them separately (Kloiber *et al*., 2013; Acikel *et al*., 2020; Chang *et al*., 2022). In our study, none of these SNPs were associated with depression treatment response at 6 weeks or 6 months after FDR-adjusting the *p*-value. One reason for that could be that the patient material was clinically different in our study compared to previous studies. First, the patients in our sample were recruited from secondary psychiatric care units. Two thirds of our patients suffered from recurrent depression. Most of them had likely been treated in primary care with antidepressants before referring them to specialised health care. Therefore, it is probable that the findings of our study reflect a prolonged state of depression, and it is most likely that our sample included more treatment-resistant patients than the average population. Second, the inclusion criteria were wider in our study than in previous studies. Unlike previous studies, we did not exclude patients with harmful alcohol use. Chronic alcohol consumption might elevate plasma leptin levels, while acute alcohol use reduces plasma leptin concentration (Bach *et al*., 2021). However, we were able to take alcohol users into account in our models, and we did our analyses also excluding patients with AUDIT scores >10, which had no substantial effect on the results.

As a secondary finding, we found an association between high leptin levels and depression severity independently of BMI. In line with our result, few studies have found an association between high leptin levels and depression severity (Cernea *et al*., 2019; Syk *et al*., 2019; Takekawa *et al*., 2019; Wittekind *et al*., 2022). One study found that high leptin levels were associated only with somatic symptoms of depression but not cognitive symptoms or all symptoms of depression (Chirinos *et al*., 2013). Morris *et al*. found that adiposity mediated the association between leptin and depression severity, and the association was not significant after controlling for BMI. In their subgroup analyses, low leptin levels associated with depression severity in normal-weight patients while in overweight patients were high leptin levels associated with more severe depression (Morris *et al*., 2012).

Previous case-control studies have yielded inconsistent results regarding the leptin levels in depression and have been limited by small sample sizes and clinical heterogeneity. Some studies have found lower leptin levels in depressed patients than in healthy controls (Kraus *et al*., 2001; Jow *et al*., 2006; Yang *et al*., 2007), while other studies have reported higher leptin levels in depressed patients than controls (Esel *et al*., 2005; Gecici *et al*., 2005; Cizza *et al*., 2012). Two latest meta-analysis did not find a significant association between leptin levels and depression (Carvalho *et al*., 2014; Cao *et al*., 2018). Large studies by Milaneschi *et al*. suggested that leptin is only associated with MDD in connection with increased neurovegetative symptoms such as appetite and weight (Milaneschi *et al*., 2017; Milaneschi *et al*., 2017). Furthermore, a previous study using the same cohort as the present study found that baseline leptin levels were higher in women, with more notable changes over a 6-month follow-up compared to men, highlighting significant gender differences (Archer *et al*., 2018).

The positive association between leptin levels and depression severity is inconsistent with leptin’s antidepressant effects observed in preclinical studies (Lu *et al*., 2006). Leptin resistance may explain this discrepancy (Lu, 2007; Yamada *et al*., 2011). The main mechanisms of leptin resistance are disorders of the blood-brain barrier transport, competitive inhibition of leptin, mutations of leptin receptor (*LEPR*), and impairment of the leptin cellular signalling (Liu *et al*., 2018). The previous data indicate that reduced leptin signalling rather than low leptin levels is associated with depression (Milaneschi *et al*., 2014). Obesity is connected to high leptin levels and the development of leptin resistance (Considine *et al*., 1996). However, there is also evidence that leptin resistance is linked to depression regardless of obesity status (Cernea *et al*., 2019).

There were certain limitations in our study. First, the sample size was relatively limited. Second, we used a Fisher’s method (*p*-values) instead of Bayesian statistics when we examined SNPs from previous studies. The real-world setting of the study was both a strength and a limitation. As mentioned earlier, the inclusion criteria were wide, and we did not exclude alcohol or other substance users. The use of antidepressants was not standardised, and the patients had different medications. In practice, patients with depression do have common alcohol and substance use and different medications, so the results are well generalisable to patient populations in secondary psychiatric health care. We were also able to take smoking, weight, and the use of alcohol into account in our models. Another limitation of our study is the absence of measurements for soluble leptin receptor levels. The inclusion of these measurements would have enabled the calculation of the Free Leptin Index, a surrogate marker of leptin resistance. Future studies should consider incorporating soluble leptin receptor levels to provide a more comprehensive analysis of leptin resistance.

In conclusion, we found an association between two *LEPR* proxy SNPs, rs12564738 and rs12029311, and depression treatment response. We also found that leptin is associated with depression severity regardless of BMI. Further studies and meta-analyses are needed to confirm the significance of these SNPs and the role of leptin in depression.
